# Hydrastis Canadensis

**Published:** 1889-02

**Authors:** A. McNeil

**Affiliations:** San Francisco, Cal.


### HYDRASTIS CANADENSIS.

A. McNeil, M. D., San Francisco, Cal.

This drug is one of the new remedies, and is an antipsoric. Its most characteristic symptoms it has in common with Kali bichrom., viz.: discharge of tough, stringy mucus from any of the mucous membranes. This mucus, like that of the Kali, may be either white or yellow, and like the bichromate, it affects all of the mucous membranes. To differentiate between these drugs we must carefully compare all the other symptoms.

Like nearly all remedies which affect the digestive organs, it has a powerful effect on the mind and disposition. The Hydrast. patient is forgetful, irritable, or despondent. It also has characteristic symptoms which show its close relation to the female generative organs. Immediately after the cessation of the menses she has a leucorrhœal discharge like the white of an egg, which continues for ten days or more and ends by becoming red and bloody; during this time, although coition is very painful, yet she has an almost constant desire, sometimes reaching a sexual fury. After this is gratified she is prostrated and distressed at the stomach, and spits up her previous meal or tastes it in her mouth. But after this discharge like the white of an egg has passed away, she becomes irritable and disposed to anger, and any reference to coition provokes her to wrath. Ammonium mur. and Bovista have a leucorrhœal discharge like the white of an egg, but neither of them have the other features of the case. There are other characteristic mental symptoms connected with the digestive and generative organs. She regurgitates her food by mouthfuls without any nausea, but if the food is not thus thrown off, she becomes despondent and gloomy, has headache, is nervous and restless. Another mental characteristic showing itself in connection with either the digestive or generative organs: she becomes nervous and irritable after dinner, and cannot bear to be spoken to, and her head aches intensely.

The characteristic discharge flows from the eyes, ears, and nose.

In the derangements of the stomach which are caused by Golden Seal, sometimes the tongue is large, showing the imprints of the teeth on its edges, precisely as with Mercurius. Sometimes the tongue feels as if it had been burned or scalded as in Sanguinaria. This remedy is indicated in the sore mouth of nursing women and of infants, when there is the characteristic mucous discharge, and more particularly if Mercury or Chlorate of Potash has been abused, and the latter drug is very often thus abused by the profession and laity alike.

In patients we sometimes find a weak, gone sensation in the pit of the stomach. If it is worse at ten A. M., it indicates Muriatic acid; at eleven A. M., Sulphur; with Sepia it is not relieved by eating but ceases after supper; with Digitalis it is so much aggravated by eating that it seems as if life would vanish; with Ignatia it is not relieved by eating, and is attended with sighing. With Oleander it is accompanied by a feeling of fullness of the abdomen, etc., is relieved by brandy; with Hydrastis it is constant and is attended by violent palpitations of the heart. This remedy is required in all gastric complaints, from simple dyspepsia to cancer, when there is vomiting of everything taken into the stomach *except milk and water mixed together.*

Give Hydrastis in constipation when the stools are hard balls covered with yellowish tough mucus. Graphites has stools of lumps connected together by threads of mucus. In hemorrhoids, when a small loss of blood is followed by excessive weakness, Hamamelis has the same condition; the choice must rest on the remaining symptoms. Hydrastis has soft stools, followed by faintness; Conium and Sarsaparilla also.

I have already described a peculiar leucorrhœa which meets its simillimum in this remedy, and the resemblance of the discharges from mucous membranes to those of Kali bichrom. Hydrastis has been lauded as the specific in cancer wherever situated. This is only partly true. There is no specific for any disease. But carcinoma, with symptoms the totality of which corresponds to Hydrastis, have been cured by it, and it is one of our cancer remedies.

In common with Lachesis and Phosphorus, it cures that condition in which small wounds bleed much.

